# Supplementary material for: Modeling glioblastoma heterogeneity as a dynamic network of cell states
Source: Mol Syst Biol. 2021 Sep 16;17(9):e10105. doi: 10.15252/msb.202010105 (PMC8444284; doi:10.15252/msb.202010105)
Supplement: Supplementary file 6 — Source Data for Figure 5 [file MSB-17-e10105-s004.zip › Figure5A_sourcedata/GSEA_3071/nosigdb_stateIII.GseaPreranked.1622795155562/DOROSZKO_U3054MG_CLUSTER_9.html]

Details for gene set DOROSZKO\_U3054MG\_CLUSTER\_9[GSEA]

|  || Dataset | state33071 |
| Phenotype | NoPhenotypeAvailable |
| Upregulated in class | na\_pos |
| GeneSet | DOROSZKO\_U3054MG\_CLUSTER\_9 |
| Enrichment Score (ES) | 0.32107276 |
| Normalized Enrichment Score (NES) | 3.8998067 |
| Nominal p-value | 0.0 |
| FDR q-value | 0.0 |
| FWER p-Value | 0.0 |
Table: GSEA Results Summary

  

Fig 1: Enrichment plot: DOROSZKO\_U3054MG\_CLUSTER\_9      
 Profile of the Running ES Score & Positions of GeneSet Members on the Rank Ordered List

  

| PROBE | GENE SYMBOL | GENE\_TITLE | RANK IN GENE LIST | RANK METRIC SCORE | RUNNING ES | CORE ENRICHMENT || 1 | ROMO1 |  |  | 14 | 0.569 | 0.0131 | Yes |
| 2 | SNRPD2 |  |  | 29 | 0.500 | 0.0242 | Yes |
| 3 | PFN1 |  |  | 40 | 0.481 | 0.0357 | Yes |
| 4 | NDUFA6 |  |  | 54 | 0.458 | 0.0458 | Yes |
| 5 | CFL2 |  |  | 74 | 0.437 | 0.0537 | Yes |
| 6 | PDCD5 |  |  | 77 | 0.435 | 0.0660 | Yes |
| 7 | OST4 |  |  | 83 | 0.429 | 0.0774 | Yes |
| 8 | MRPL52 |  |  | 99 | 0.411 | 0.0855 | Yes |
| 9 | ATP5MD |  |  | 106 | 0.407 | 0.0960 | Yes |
| 10 | COX5A |  |  | 131 | 0.389 | 0.1012 | Yes |
| 11 | DNAJC8 |  |  | 136 | 0.384 | 0.1114 | Yes |
| 12 | SUGT1 |  |  | 185 | 0.349 | 0.1092 | Yes |
| 13 | PSME1 |  |  | 190 | 0.347 | 0.1184 | Yes |
| 14 | ATP5PF |  |  | 191 | 0.346 | 0.1286 | Yes |
| 15 | NDUFB6 |  |  | 199 | 0.339 | 0.1367 | Yes |
| 16 | PFDN2 |  |  | 202 | 0.338 | 0.1461 | Yes |
| 17 | ATP5MC1 |  |  | 208 | 0.334 | 0.1546 | Yes |
| 18 | SAP18 |  |  | 238 | 0.315 | 0.1564 | Yes |
| 19 | MRPS18C |  |  | 251 | 0.310 | 0.1624 | Yes |
| 20 | PTGES3 |  |  | 252 | 0.310 | 0.1715 | Yes |
| 21 | IMPDH2 |  |  | 253 | 0.309 | 0.1806 | Yes |
| 22 | ELOC |  |  | 271 | 0.302 | 0.1850 | Yes |
| 23 | NDUFB9 |  |  | 278 | 0.297 | 0.1922 | Yes |
| 24 | ARF1 |  |  | 281 | 0.296 | 0.2004 | Yes |
| 25 | ATP5MC2 |  |  | 282 | 0.296 | 0.2091 | Yes |
| 26 | CDC42 |  |  | 289 | 0.295 | 0.2162 | Yes |
| 27 | EIF3H |  |  | 291 | 0.294 | 0.2246 | Yes |
| 28 | MYL9 |  |  | 293 | 0.293 | 0.2330 | Yes |
| 29 | SDHB |  |  | 323 | 0.286 | 0.2338 | Yes |
| 30 | ATP5MC3 |  |  | 334 | 0.283 | 0.2395 | Yes |
| 31 | AIMP1 |  |  | 336 | 0.282 | 0.2476 | Yes |
| 32 | SH3GLB1 |  |  | 338 | 0.281 | 0.2556 | Yes |
| 33 | ARF4 |  |  | 348 | 0.277 | 0.2614 | Yes |
| 34 | PFDN6 |  |  | 352 | 0.274 | 0.2687 | Yes |
| 35 | CHMP4B |  |  | 353 | 0.274 | 0.2768 | Yes |
| 36 | DNAJA1 |  |  | 355 | 0.273 | 0.2846 | Yes |
| 37 | TOP1 |  |  | 382 | 0.267 | 0.2856 | Yes |
| 38 | ZCRB1 |  |  | 395 | 0.261 | 0.2902 | Yes |
| 39 | TAGLN |  |  | 407 | 0.257 | 0.2949 | Yes |
| 40 | EIF3M |  |  | 419 | 0.253 | 0.2995 | Yes |
| 41 | SERBP1 |  |  | 420 | 0.253 | 0.3069 | Yes |
| 42 | CWC15 |  |  | 422 | 0.253 | 0.3141 | Yes |
| 43 | PITPNC1 |  |  | 438 | -0.251 | 0.3176 | Yes |
| 44 | FILIP1L |  |  | 454 | -0.252 | 0.3211 | Yes |
| 45 | REL |  |  | 530 | -0.256 | 0.3091 | No |
| 46 | TM9SF2 |  |  | 664 | -0.263 | 0.2821 | No |
| 47 | TRPS1 |  |  | 690 | -0.264 | 0.2834 | No |
| 48 | IGF2BP3 |  |  | 754 | -0.267 | 0.2748 | No |
| 49 | KMT2A |  |  | 773 | -0.268 | 0.2780 | No |
| 50 | DNAJC3 |  |  | 780 | -0.268 | 0.2844 | No |
| 51 | EPRS |  |  | 805 | -0.270 | 0.2860 | No |
| 52 | KLF4 |  |  | 973 | -0.278 | 0.2507 | No |
| 53 | ASAP1 |  |  | 1002 | -0.280 | 0.2516 | No |
| 54 | PMP22 |  |  | 1023 | -0.281 | 0.2547 | No |
| 55 | PRRX1 |  |  | 1032 | -0.281 | 0.2609 | No |
| 56 | NPTN |  |  | 1054 | -0.282 | 0.2637 | No |
| 57 | SLC38A2 |  |  | 1055 | -0.282 | 0.2720 | No |
| 58 | WLS |  |  | 1517 | -0.307 | 0.1609 | No |
| 59 | TOB1 |  |  | 1573 | -0.310 | 0.1557 | No |
| 60 | SPRY2 |  |  | 1592 | -0.311 | 0.1602 | No |
| 61 | NFIX |  |  | 1848 | -0.327 | 0.1034 | No |
| 62 | ZIC1 |  |  | 2062 | -0.339 | 0.0579 | No |
| 63 | ZNF90 |  |  | 2220 | -0.350 | 0.0273 | No |
| 64 | SASH1 |  |  | 2231 | -0.351 | 0.0350 | No |
| 65 | FBXW7 |  |  | 2258 | -0.353 | 0.0386 | No |
| 66 | NEO1 |  |  | 2290 | -0.356 | 0.0410 | No |
| 67 | IQGAP1 |  |  | 2405 | -0.366 | 0.0221 | No |
| 68 | SDC2 |  |  | 2537 | -0.376 | -0.0010 | No |
| 69 | SPTBN1 |  |  | 2647 | -0.385 | -0.0180 | No |
| 70 | ACTA2 |  |  | 2721 | -0.393 | -0.0255 | No |
| 71 | ADAMTS1 |  |  | 2736 | -0.394 | -0.0176 | No |
| 72 | JMY |  |  | 2740 | -0.394 | -0.0068 | No |
| 73 | PRR7 |  |  | 2779 | -0.397 | -0.0050 | No |
| 74 | LZTS2 |  |  | 2847 | -0.403 | -0.0106 | No |
| 75 | ATP1B1 |  |  | 2865 | -0.405 | -0.0031 | No |
| 76 | FURIN |  |  | 2932 | -0.412 | -0.0082 | No |
| 77 | JUN |  |  | 2935 | -0.413 | 0.0035 | No |
| 78 | PAM |  |  | 3043 | -0.425 | -0.0119 | No |
| 79 | CHPT1 |  |  | 3060 | -0.426 | -0.0035 | No |
| 80 | PLEKHH3 |  |  | 3076 | -0.428 | 0.0052 | No |
| 81 | ARIH1 |  |  | 3098 | -0.431 | 0.0124 | No |
| 82 | HSPA5 |  |  | 3193 | -0.442 | 0.0009 | No |
| 83 | KCTD1 |  |  | 3310 | -0.462 | -0.0157 | No |
| 84 | ITM2B |  |  | 3314 | -0.463 | -0.0029 | No |
| 85 | IP6K1 |  |  | 3407 | -0.481 | -0.0127 | No |
| 86 | JAG1 |  |  | 3424 | -0.486 | -0.0025 | No |
| 87 | KDM6A |  |  | 3476 | -0.500 | -0.0011 | No |
| 88 | ZFP36L1 |  |  | 3487 | -0.503 | 0.0111 | No |
| 89 | ITGB1 |  |  | 3574 | -0.537 | 0.0045 | No |
| 90 | APLP2 |  |  | 3693 | -0.610 | -0.0083 | No |
| 91 | CYR61 |  |  | 3887 | -1.037 | -0.0281 | No |
| 92 | NFIA |  |  | 3910 | -1.317 | 0.0050 | No |
Table: GSEA details [plain text format]

  

Fig 2: DOROSZKO\_U3054MG\_CLUSTER\_9: Random ES distribution      
 Gene set null distribution of ES for **DOROSZKO\_U3054MG\_CLUSTER\_9**
